# Enhancer RNA LINC00242-Induced Expression of PHF10 Drives a Better Prognosis in Pancreatic Adenocarcinoma

**DOI:** 10.3389/fonc.2021.795090

**Published:** 2022-01-20

**Authors:** Wen Tong, Liuyang Zhu, Yi Bai, Long Yang, Zirong Liu, Yamin Zhang

**Affiliations:** ^1^ Tianjin First Central Hospital Clinic Institute, Tianjin Medical University, Tianjin, China; ^2^ Department of Hepatobiliary Surgery, Tianjin First Central Hospital, School of Medicine, Nankai University, Tianjin, China

**Keywords:** enhancer RNA, LINC00242, PHF10, pancreatic cancer, biomarker

## Abstract

Enhancer RNA is a kind of non-coding RNA, which is transcribed from the enhancer region of gene and plays an important role in gene transcription regulation. However, the role of eRNA in pancreatic adenocarcinoma (PAAD) is still unclear. In this study, we identified the key eRNA and its target gene in PAAD. The transcriptome data and clinical information of pancreatic cancer were downloaded from the UCSC Xena platform. A total of 2,695 eRNAs and its target gene predicted by the PreSTIGE method were selected as candidate eRNA–target pairs. After survival analysis, we found that LINC00242 was the eRNA most related to patients’ survival, and correlation analysis further indicated that LINC00242 and its target gene PHF10 had a significant co-expression relationship. Downregulation of LINC00242 was significantly associated with unfavorable clinicopathological features. Based on pan-cancer analysis, we found that LINC00242 was associated with the survival of multiple cancers, and LINC00242 was co-expressed with its target genes in multiple cancer types. External experiments further demonstrated that PHF10 was the downstream target gene of LINC00242. After ssGSEA analysis, PAAD patients were classified as high, medium, and low immune cell infiltration clusters. Compared with the low and medium immune infiltration clusters, the expression level of PHF10 was significantly upregulated in the high immune infiltration clusters. After performing the CIBERSORT algorithm, we found that there was a significant difference in the abundance of immune infiltrating cells between the PHF10 high- and low-expression groups. Additionally, the web tool TIMER was used to detect the distribution and expression of PHF10 in pan-cancer.

## Introduction

According to reports, pancreatic adenocarcinoma ranks fourth in cancer-related deaths, with a 5-year survival rate of approximately 9% (1). Up to now, surgical resection is still the only effective treatment in theory. However, pancreatic adenocarcinoma tends to be asymptomatic in its early stages, and most patients with PAAD are not suitable for surgical treatment when they are diagnosed in the late stage (2). In recent years, studies have confirmed that several treatment methods including immunotherapy (3, 4) and targeted therapy (5, 6) can improve the survival rate of PAAD patients, but the effect is still limited. This may be related to the different sensitivity caused by the complexity and heterogeneity of tumor tissue. Therefore, there is an urgent need to find biomarkers related to the prognosis of PAAD and to predict the sensitivity of immunotherapy and targeted therapy.

Previous studies have shown that active enhancers are transcribed and produce a series of non-coding RNAs called enhancer RNA (eRNA) (7–9). Some research suggests that eRNAs interact with RNA polymerase to promote the formation of the promoter–enhancer loop and act as a significant role in the regulation of gene expression (10, 11). The production of eRNA is a widespread phenomenon, which is related to the regulation of gene expression in a variety of cells. It has been confirmed that the transcriptional regulation process involved in eRNAs was associated with tumorigenesis and progression (12, 13). Elodie Bal reported that mutations in enhancer RNA elements may weaken the activity of enhancers and lead to abnormal activation of Hedgehog signaling pathways, thus promoting tumor development (14). In addition, Pan found that the abnormal expression of eRNA in prostate cancer was associated with cancer progression (15).

In this study, we found that eRNA LINC00242 transcribed from the enhancer region of PHF10 was significantly correlated with patients’ survival. The downregulation of LINC00242 expression was related with poor clinicopathological features. Then, we further analyzed the expression of PHF10 in pancreatic cancer and its prognostic value. Additionally, the web tool Tumor Immune Estimation Resource (TIMER) was used to study the expression level of PHF10 in pan-cancer. In addition, we also explored the relationship between the expression of PHF10 and immune infiltration. By running the single-sample gene set enrichment analysis (ssGSEA) algorithm, we divided PAAD patients into high, medium, and low immune clusters. Then, we investigate the relationship between PHF10 and three immune clusters. In order to evaluate the effect of PHF10 on the tumor microenvironment, we analyzed the tumor-infiltrating immune cells (TIICs) associated with PHF10 expression by CIBERSORT on the basis of TCGA cohort.

## Materials and Methods

### Data Acquisition and Identification of eRNAs Related to the Prognosis of PAAD

The transcriptome data and clinical information about PAAD and 32 other cancer types were downloaded from the UCSC website (https://xena.ucsc.edu/). The PAAD data set from UCSC was selected as the training set. We select ICGC-PAAD (N= 182) and GSE15471 (N = 36) as the validation set. ICGC-PAAD (N = 182) included 182 pancreatic cancer samples from different patients. GSE15471 (N = 36) included 36 pairs of pancreatic cancer and normal tissue samples. The full clinical information about these two cohorts is shown in **Supplementary Table 4**.

Based on previous studies, we obtained a list of eRNAs and its target genes predicted by the PresSTIGE method (16, 17). In the training set, the patients’ eRNA transcriptional data and clinical data were matched together. We used R software package “survival” and “survminer” to investigate the relationship between eRNAs and OS in PAAD patients. The correlation level of eRNAs and its target gene was evaluated through co-expression analysis methods. The most significant survival-associated eRNA and its target gene were selected for further analysis.

### Comprehensive Analysis of LINC00242 and PHF10

Based on the website Human Protein Atlas (HPA, http://www.proteinatlas.org/) (18, 19), we explored the mRNA and protein levels of PHF10 in various cancer tissues and normal tissues. Meanwhile, we downloaded the immunohistochemical images of PHF10 protein in pancreatic cancer from this website. The differential mRNA expression level of LINC00242 and PHF10 between PAAD tissues and normal tissues were analyzed *via* the Gene Expression Profiling Interactive Analysis (GEPIA) web tool (http://gepia.cancer-pku.cn/) (20). GSE15471 was selected to verify the difference in the expression of LINC00242 and PHF10 between PAAD tissues and normal tissues. Through univariate and multivariate Cox regression analyses, we determined the independent prognostic value of LINC00242. The correlation between LINC00242 expression level and clinicopathological was explored. ICGC-PAAD was selected to verify the relationship between LINC00242 expression and clinical parameter. Besides, we selected the differentially expressed genes in tumor compared with normal tissues and co-expressed genes to do the GO and KEGG analyses. Next, the prognostic value of LINC00242 and its co-expression relationship with PHF10 were verified in TCGA (32 other types of cancer) pan-cancer data.

### Cell Lines and Transfection

The human pancreatic cancer cell line (PANC-1) was purchased from the Cell Bank of the Chinese Academy of Sciences (Shanghai, China). We use DMEM medium supplemented with 10% fetal bovine serum (Thermo Fisher Scientific, USA) and 1% penicillin–streptomycin to culture the cells at 37°C, 5% CO2 atmosphere. LINC00242 siRNAs were synthesized by GeneChem (Shanghai, China), and Lipofectamine 2000 (Invitrogen, California, USA) was employed to achieve the cell transfection.

### The Relationship Between LINC00242 and PHF10 Was Verified by qPCR

Differently treated PAAD cell lines were collected in the logarithmic growth phase and mixed with 1 ml TRIzol. Chloroform was added and kept at room temperature for 15 min, then isopropanol was added to the solution and centrifuged to obtain RNA precipitate. qRT-PCR analyses of LINC00242 and PHF10 were performed as described previously. The transcription level was calculated by the 2^−ΔΔCt^ method. GAPDH was used as an internal reference gene. The primer sequences of LINC00242 were 5*’*-GCGGGAAGATTTCAGGCGCTTT-3*’* (forward) and 5*’-*CAGGTGGTGAAGTGAGGAACAG-3*’* (reverse). The primer sequences of PHF10 were 5*’*-GCACTCTAGGCTTAACAGCATT (forward) and 5*’-*AGCATGTTTGGCTGGATATTCTT-3*’* (reverse). The primer sequences of GAPDH were 5*’*-GTCTCCTCTGACTTCAACAGCG-3*’* (forward) and 5*’-*ACCACCCTGTTGCTGTAGCCAA-3*’* (reverse).

### Analysis of Immune Cell Patterns in the Microenvironment

The abundance of 22 immune cells in UCSC-PAAD and ICGC-PAAD was estimated *via* the CIBERSOFT website (https://cibersort.stanford.edu/index.php) (21). Monte Carlo sampling was used to calculate the p value. Only samples with CIBERSORT p < 0.05 were included in the further study. Based on the median value of PHF10 expression, we divided PAAD patients into a high-expression group (PHF10-H) and a low-expression group (PHF10-L). We used violin diagrams to show the difference in the content of 22 immune cells between PHF10-H and PHF10-L groups. From the previous studies, we obtained the immune-related gene set with 29 immune cell types and immune-related functions (22). Then, we used the ssGSEA algorithm to obtain the enrichment score with the “GSVA” package in R (23). PAAD samples from UCSC-PAAD and ICGC-PAAD were divided into three immune subgroups by the unsupervised clustering method based on ssGSEA scores. In order to further explore the difference of the immune microenvironment between three immune subgroups, the “estimate” package in R (24) was used to calculate the immune score, stromal score, and ESTIMATE score. Next, we use the “pheatmap” package in R to visualize the results. Additionally, the expression levels of PHF10 and the human leukocyte antigen (HLA) gene family were compared between three immune subgroups.

### Intergrated Analysis of PHF10 Expression and Immune Cell Infiltration and Immune Subtype in Pan-Cancer

The Tumor Immune Estimation Resource (TIMER, https://cistrome.shinyapps.io/timer/) website (25) was used to evaluate the relationship between the expression level of PHF10 and immune cell infiltration. TIMER applies a statistical method called deconvolution to infer the abundance of TIICs according to RNA sequencing data. The relationship between immune subtype and mRNA level of PHF10 was explored *via* tumor–immune system interactions and DrugBank (TISIDB, http://cis.hku.hk/TISIDB/index.php) (26) database by the Subtype module.

### Statistical Analysis

All statistical analyses were operated *via* R software (version 4.0.3), GraphPad Prism (version 8.0), and IBM SPSS Statistics (version 25.0). The correlations between PHF10 and clinicopathological features were analyzed by the χ2 test. Univariate and multivariate analyses were used to determine prognostic factors. Spearman rank correlation analysis was used to estimate the correlation strength. Kaplan–Meier analysis was used to compare the survival rate among different groups. Unless noted otherwise, p < 0.05 was considered statistically significant.

## Results

### Prognostic Value of eRNAs in PAAD

**Figure 1:**
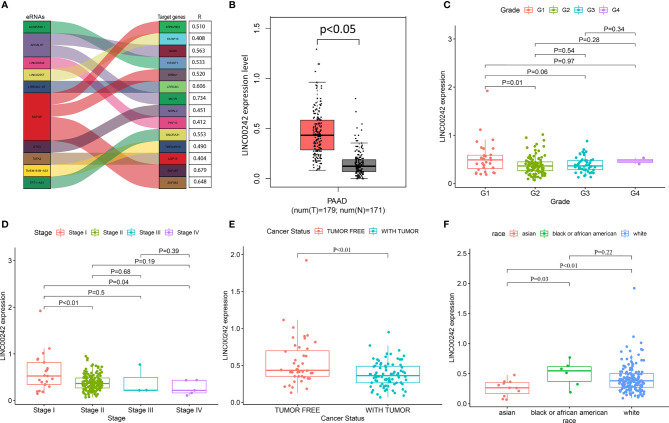
The correlation analysis between LINC00242 and the clinicopathological characteristics of PAAD. **(A)** Top 10 eRNAs and their target genes most relevant to survival. **(B)** LINC00242 expression was significantly upregulated in PAAD samples compared with normal samples. **(C, D)** LINC00242 expression was negatively correlated with histological grade **(C)** and AJCC stage **(D)**. **(E)** Tumor status had a low expression level of LINC00242 compared with tumor-free status. **(F)** The expression level of LINC00242 in Asian patients was significantly lower than that in the other two races.

After removing samples with missing values, a total of 177 patients were included in the study. The clinicopathological characteristics of the patients are shown in **Supplementary Table 1**. We obtained 2,695 eRNA transcripts and 2,303 predicted target genes from previous studies. Next, the 2,695 eRNA transcript IDs were mapped to their corresponding 1,559 genes based on the Ensembl Gene ID from the Ensembl database (http://asia.ensembl.org/info/data/ftp/index.html). According to gene expression profile and clinical information from 177 PAAD patients, we identified 15 of the 1,559 candidate eRNAs, which were significantly correlated with patients’ OS (Kaplan–Meier, p < 0.01; **Supplementary Table 2**). Then, we found that all 15 eRNAs were significantly correlated with their target genes (R > 0.4, p < 0.01; **Supplementary Table 2**). The relationship between top 10 eRNAs associated with survival and its target gene is shown in **Figure 1A**.

### LINC00242 Is a Key eRNA in PAAD

LINC00242 is the most survival related eRNA, and it is significantly positively correlated with the predicted target PHF10 (**Figure 1A** and **Supplementary Table 2**). Then, LINC00242 and its target PHF10 were selected for further analysis. According to the gene expression data of 179 PAAD tissues and 171 normal pancreatic tissues from the TCGA and GTEx databases, the mRNA level of LINC00242 was significantly upregulated in PAAD samples (**Figure 1B**, p < 0.05). However, there was no significant difference in LINC00242 expression between pancreatic carcinoma and normal tissues in our validation set (**Supplementary Figure 3C**). The expression level of LINC00242 was significantly correlated with some clinical features of PAAD, including cancer status (p = 0.005), race (p = 0.02), and history of chronic pancreatitis (p = 0.004) (**Supplementary Table 1**). Compared with G1, the expression level of LINC00242 of G2 was lower (**Figure 1C**, p < 0.05). In addition, patients with advanced tumor stage were also correlated with low expression level of LINC00242 (**Figure 1D**, p < 0.05). Furthermore, cancer status also associated with LINC00242 expression level. Tumor free status had higher gene expression level when compared with tumor status (**Figure 1E**, p < 0.05). Interestingly, we found that patients of Asian descent had lower gene expression than patients of the other two races, including black or African American and white (**Figure 1F**, p < 0.05).

**Figure 2:**
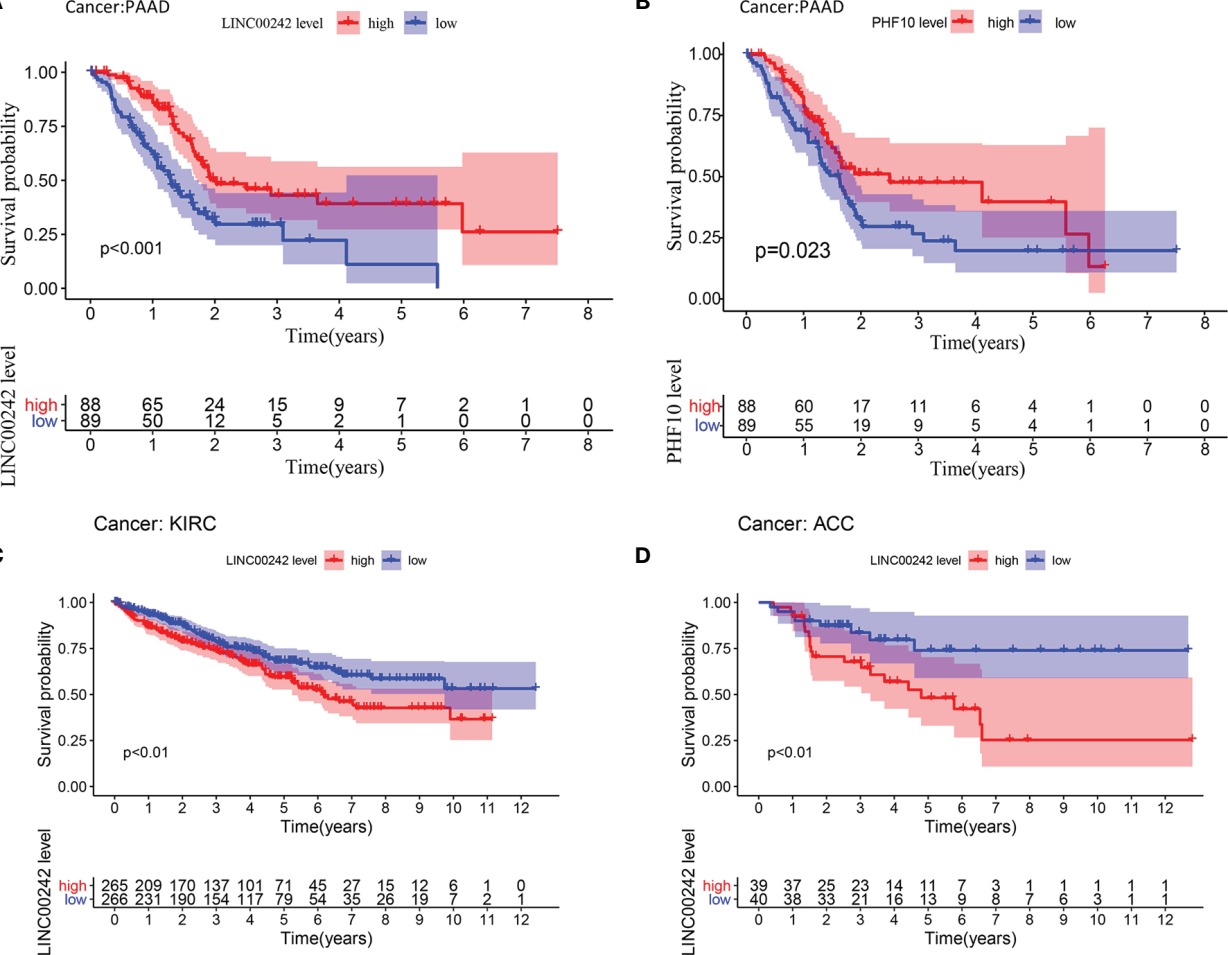
Survival analysis of LINC00242 and its target gene PHF10. **(A)** LINC00242 upregulation was significantly correlated with better OS in PAAD. **(B)** PHF10 upregulation was significantly correlated with better OS in PAAD. **(C)** LINC00242 upregulation was significantly correlated with worse OS in KIRC. **(D)** LINC00242 upregulation was significantly correlated with worse OS in ACC. PAAD, pancreatic adenocarcinoma; KIRC, kidney renal clear cell carcinoma; ACC, adrenocortical carcinoma.

Based on the median expression level of LINC00242, we divided 177 patients into high- and low-expression groups. High-expression LINC00242 was significantly correlated with better OS (Kaplan–Meier, p < 0.01, **Figure 2A**). Unfortunately, we did not find a significant relationship between LINC00242 expression and prognosis as well as clinical parameters (TNM stage, grade, data not displayed) in ICGC-PAAD (N = 182), which may be related to the low abundance of non-coding RNA and the different sequencing methods (**Supplementary Figure 3A**). High PHF10 expression was also associated with better OS (p < 0.01, **Figure 2B**). The analysis based on ICGC-PAAD showed that high expression of PHF10 was closely related to the better prognosis of the patients (**Supplementary Figure 3B**). What is more, the prognostic value of LINC00242 was explored in other cancer types from the TCGA database. LINC00242 also plays a prognostic role in KIRC and ACC. A high expression level of LINC00242 was significantly associated with poor OS in KIRC and ACC (p < 0.01, **Figures 2C, D** and **Supplementary Table 3**).

**Figure 3:**
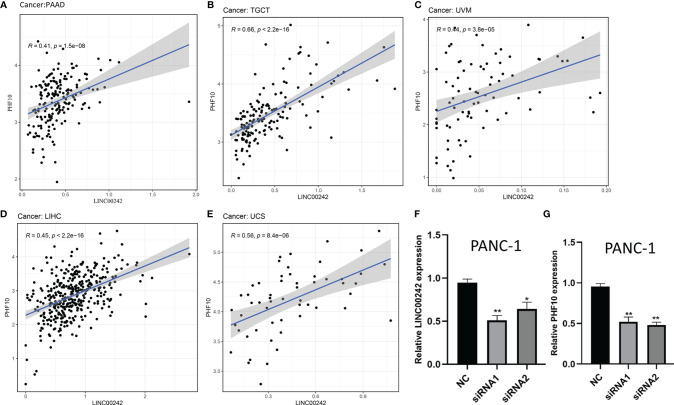
The correlation analysis between LINC00242 and its target PHF10 in pan-cancer. **(A–E)** A significant co-expression relationship between LINC00242 and PHF10 was observed in PAAD, TCGT, UVM, LIHC, and UCS. **(F)** The expression level of LINC00242 were significantly downregulated after being transfected with LINC00242 siRNA. **(G)** The expression level of PHF10 was significantly downregulated after transfected with LINC00242 siRNA. TCGT, testicular germ cell tumors; UVM, uveal melanoma; LIHC, liver hepatocellular carcinoma; UCS, uterine carcinosarcoma (*p < 0.05, **p < 0.01).

In addition, we found a significant co-expression correlation between LINC00242 and its target gene PHF10 in multiple cancer types, including PAAD, KIRC, ACC, TGCT, UVM, LIHC, and UCS (p < 0.01, **Figures 3A–E** and **Supplementary Table 3**). To further verify the regulatory relationship between LINC00242 and PHF10, we knocked down the expression of LINC00242 with transfection of LINC00242 siRNA. We used qRT-PCR to examine the efficiency of knockdown. As depicted in **Figure 3F**, the expression of LINC00242 was significantly downregulated after si-LINC00242 transfection (p < 0.05, **Figure 3F**). Of note, compared with the si-control group, the expression of PHF10 was significantly downregulated in si-LINC00242 group (p < 0.05, **Figure 3G**).

### Independent Prognostic Value of LINC00242 in PAAD

**Figure 4:**
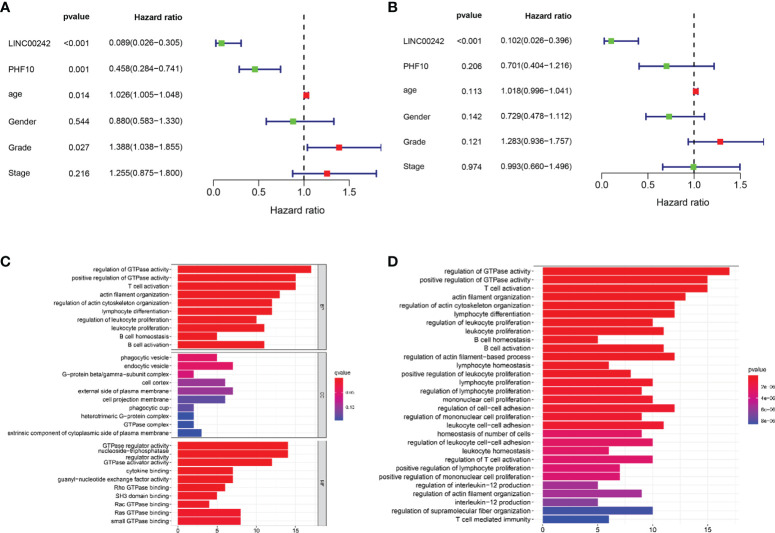
Forest plot of Cox regression analysis and functional enrichment analysis in PAAD. **(A)** Univariate Cox regression analysis. **(B)** Multivariate Cox regression analysis. **(C, D)** The biological processes of Gene Ontology (GO) analysis **(C)** and enrichment pathway of Kyoto Encyclopedia of Genes and Genomes (KEGG) analysis **(D)**.

The univariate and multivariate Cox analyses were used to estimate the prognostic value of LINC00242 in PAAD. The univariate analysis showed that age (p < 0.05), grade (p < 0.05), PHF10 (p < 0.01), and LINC00242 (p < 0.01) were significantly associated with OS (**Figure 4A**). In the multivariate analysis, LINC00242 (p < 0.01) was still an independent prognostic factor of OS (**Figure 4B**).

### Functional Enrichment Analysis

Through the intersection of differentially expressed genes and co-expressed genes, we identified a total of 103 genes. GO and KEGG enrichment analyses showed that these genes were involved in many kinds of immune-related pathways and signaling pathways, such as T cell activation and lymphocyte differentiation (**Figures 4C, D**).

### mRNA and Protein Expression Profile of PHF10

**Figure 5:**
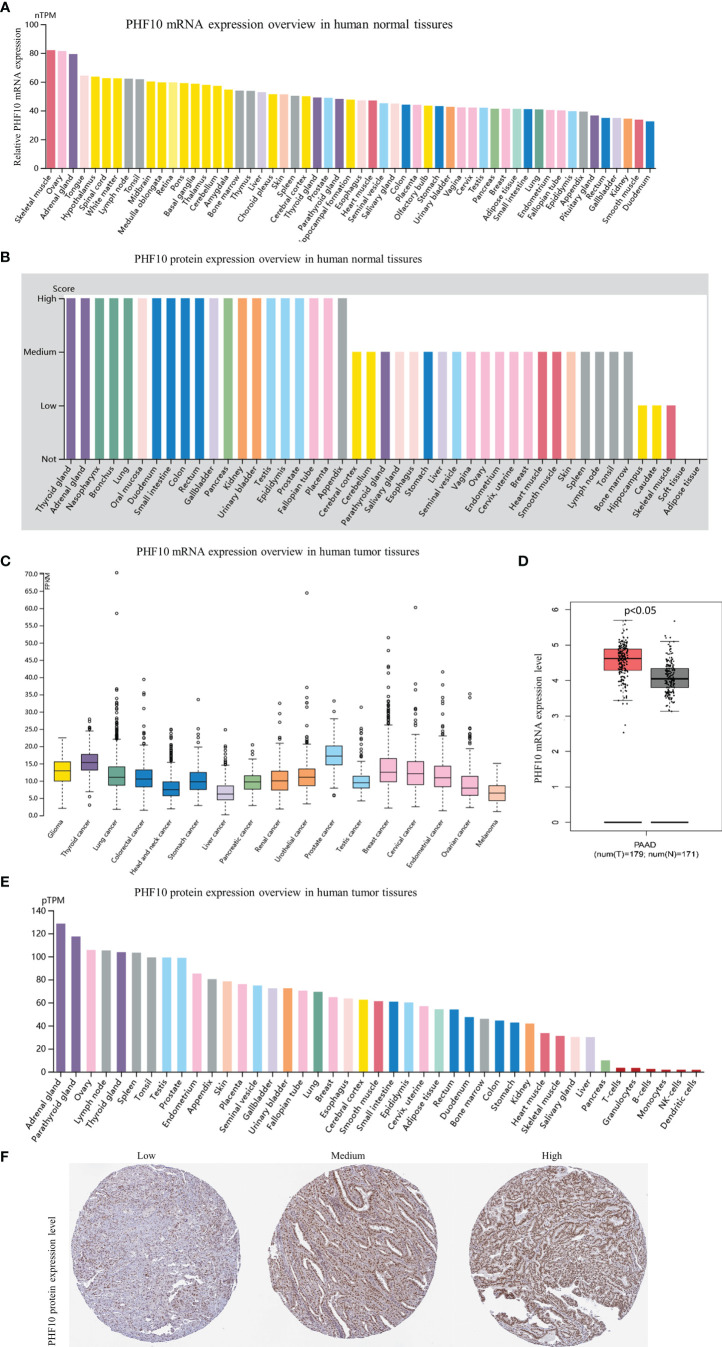
Gene and protein expression profiles of PHF10 in tumor tissues and normal tissues. **(A)** PHF10 mRNA expression overview in human normal tissues. **(B)** PHF10 protein expression overview in human normal tissues. **(C)** PHF10 mRNA expression overview in human tumor tissues. **(D)** Comparison of PHF10 mRNA expression between pancreatic cancer tissues and normal tissues. **(E)** PHF10 protein expression overview in human tumor tissues. **(F)** Representative immunohistochemistry (IHC) images pictures with PHF10 antibody (1:50, Cat#HPA055649, Sigma-Aldrich). All data were obtained from the HPA database (https://www.proteinatlas.org/).

The HPA website was used to analyze the expression level of PHF10 in different tissues. The RNA expression of PHF10 in normal pancreatic tissue was relatively low (**Figure 5A**), but the protein level of PHF10 was relatively high among 43 normal human tissue types (**Figure 5B**). Additionally, the PHF10 mRNA level in PAAD samples was relatively low among 17 cancer types (**Figure 5C**); However, the expression of PHF10 was significantly upregulated in PAAD compared with normal tissues (**Figure 5D**). Based on GSE15471 analysis, the expression of PHF10 in pancreatic cancer tissues was significantly higher than that in normal tissues (**Supplementary Figure 3D**). In terms of the expression level of PHF10 protein, pancreatic cancer showed a low positive rate, ranking seventh from the bottom among 43 common cancer types (**Figure 5E**). The representative IHC images with different PHF10 expression levels, including low, medium, and high, are shown in **Figure 5F**. Based on web tool TIMER, we further examined PHF10 expression in multiple human cancers with RNA-seq data from the TCGA database. Interestingly, the PHF10 expression level varied significantly among different cancer types. PHF10 expression was significantly upregulated in CHOL, COAD, LIHC, and STAD and significantly downregulated in BLCA, BRCA, KICH, KIRC, KIRP, LUAD, THCA, and UCEC, than in normal tissues (**Supplementary Figure 1**).

### Integrated Analysis of PHF10 Expression and Immune Infiltration in PAAD

**Figure 6:**
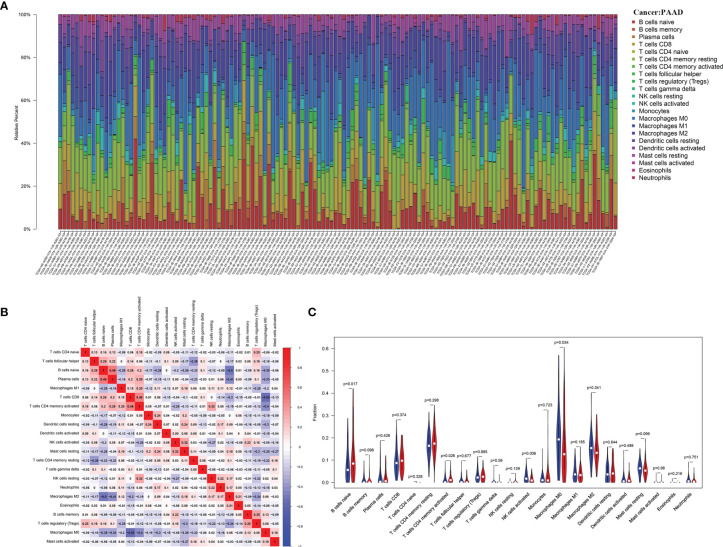
Correlation’s analysis of PHF10 expression with immune infiltration calculated by the CIBERSORT algorithm in the TCGA cohort. **(A)** The landscape of immune infiltration in 133 pancreatic cancer tissues. **(B)** Correlation analysis of different types of immune cell infiltration. **(C)** Analysis of the difference of immune cell abundance between the low and high PHF10 expression group.

After performing the CIBERSORT algorithm, 134 tumor samples with p< 0.05 in the TCGA cohort were enrolled in this study. The landscape of immune infiltrations consisted by 22 immune cells is shown in **Figure 6A**. There was a significant positive correlation between activated CD4^+^ memory T cells and CD8^+^ T cells, and a significant negative correlation between CD8^+^ T cells and M0 macrophage (**Figure 6B**). To further investigate the effect of PHF10 on TIICs, all PAAD samples were assigned into high-expression (PHF10-H) and low-expression (PHF10-L) groups based on the median expression level of PHF10. Obviously, there was a significant difference in the proportion of TIICs between the two groups (**Figure 6C**). Compared with the PHF10-H group, the PHF10-L group contained a higher proportion of activated NK cells and M0 macrophages, but the proportion of naive B cells and resting CD4^+^ memory T cells was relatively lower (all p < 0.05, **Figure 6C**).

**Figure 7:**
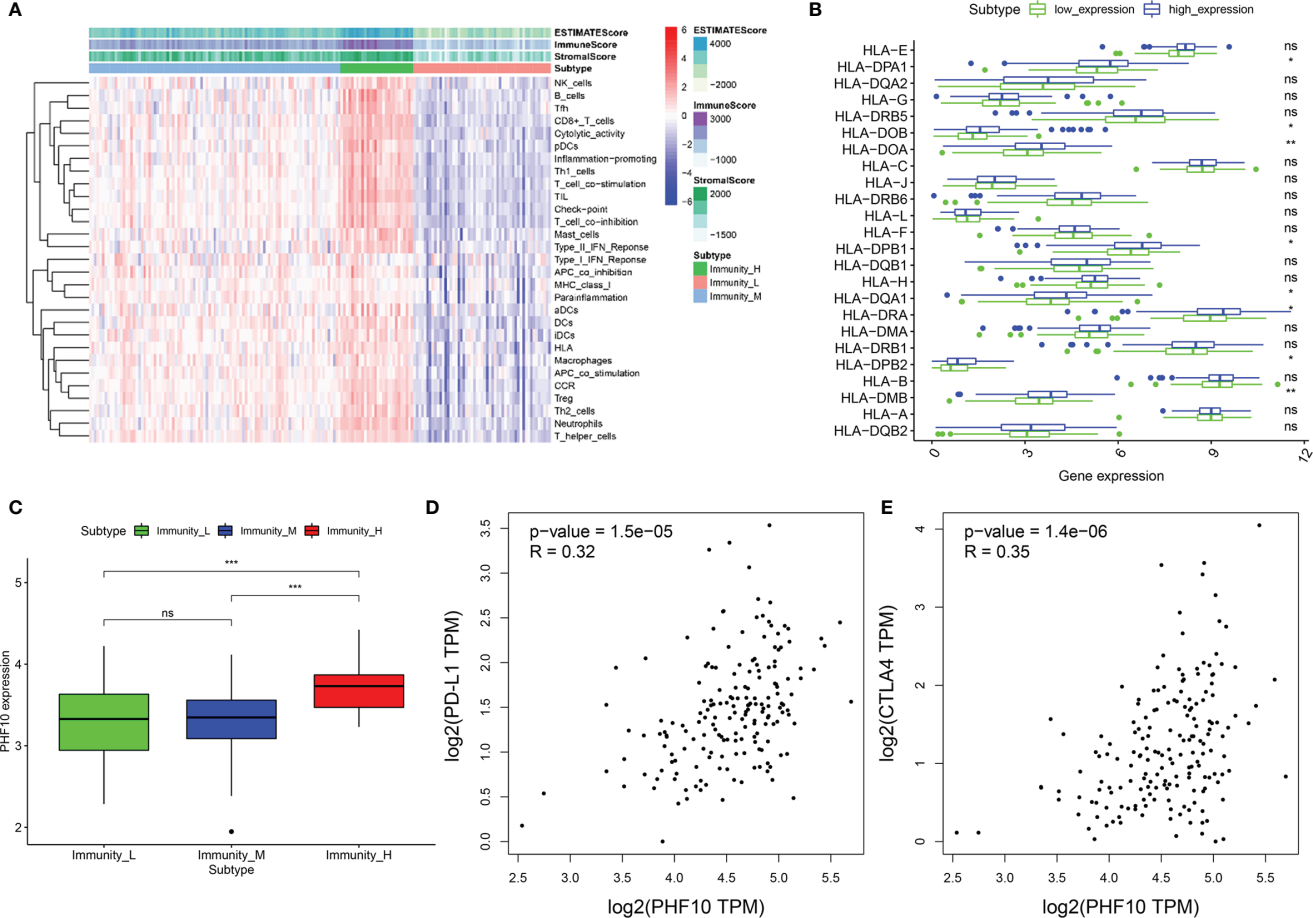
The integrated analysis of PHF10 with immune infiltration. **(A)** The enrichment levels of 29 immune-related gene sets were calculated *via* the ssGSEA method. **(B)** The expression of the HLA gene family in the PHF10 high-expression group was significantly different with that of the PHF10 low-expression group. **(C)** The expression of PHF10 varied significantly among the three clusters, which were obtained by the unsupervised clustering method based on ssGSEA results. **(D, E)** Co-expression analysis of PHF10 and immune checkpoint genes PD-L1 and CTLA4. (*p < 0.05, **p < 0.01, ***p < 0.001. ns, no statistically significant).

We used the ssGSEA algorithm to obtain the enrichment score. Using an unsupervised clustering algorithm, PAAD samples were assigned into three immune infiltration clusters, including high immune cell infiltration cluster (Immunity-H, n = 97), medium immune cell infiltration cluster (Immunity-M, n = 28), and low immune cell infiltration cluster (Immunity-L, n = 53). Then, we calculated the Immune score, Stromal score, and ESTIMATE score based on the normalized gene expression data. The Immune score, Stromal score, and ESTIMATE score of the Immunity-H cluster are higher than the other two clusters (**Figure 7A**). The box plot also showed that with the increase in PHF10 expression, the expression levels of several HLA related genes were upregulated (**Figure 7B**). Additionally, the expression level of PHF10 in the Immunity-H cluster was significantly upregulated compared with the Immunity-M and Immunity-L clusters (all p< 0.05, **Figure 7C**). Based on ssGESA analysis, pancreatic cancer patients in the ICGC-PAAD cohort can be divided into three categories, which was consistent with the results of the training set (**Supplementary Figure 4A**). In addition, there was infiltration of more B cell-naive and fewer M0 macrophages in the PHF10 high-expression group, which was consistent with the training set (**Supplementary Figure 4B**). At the same time, the expression of PHF10 in the Immunity-H group was significantly higher than that in the Immunity-M group (**Supplementary Figure 4C**).

Concomitantly, we further investigate the relationships between PHF10 expression and immune checkpoint genes (PD-L1 and CTLA-4) in PAAD *via* GEPIA. The results demonstrated that PHF10 was positively correlated with the expression of PD-L1 (R = 0.32, p < 0.01, **Figure 7D**) and CTLA4 (R = 0.35, p < 0.01, **Figure 7E**).

### Pan-Cancer Analysis of the Association of PHF10 With Immune Subtypes and Immune Cell Infiltration

**Figure 8:**
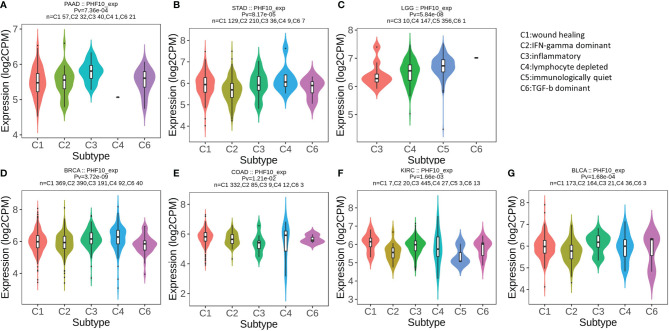
Correlation analysis of PHF10 expression with immune subtype in pan-cancer. **(A–G)** Correlation of PHF10 expression and immune subtypes in PAAD, STAD, LGG, BRCA, COAD, KIRC, and BLCA. STAD, stomach adenocarcinoma; LGG, brain lower grade glioma; BRCA, breast invasive carcinoma; COAD, colon adenocarcinoma; BLCA, bladder urothelial carcinoma.

**Figure 9:**
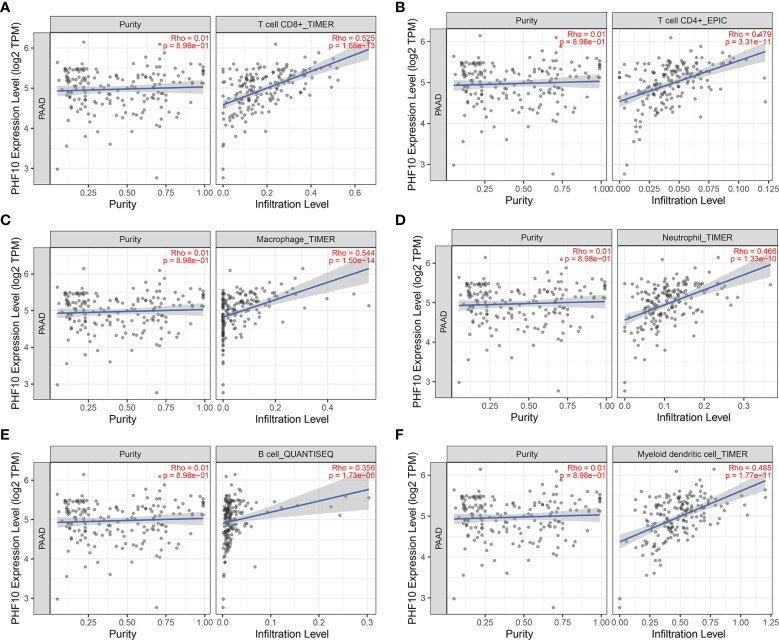
Correlation analysis between PHF10 expression and immune cell infiltration in PAAD. **(A–F)** The expression of PHF10 was positively correlated with the infiltration of immune cells.

Then, we use the TISIDB database to explore whether PHF10 was correlated with the immune subtype of PAAD. All tumor samples in the TISIDB database were divided into six immune subtypes, including C1: wound healing, C2: IFN-gamma dominant, C3: inflammatory, C4: lymphocyte depleted, C5: immunologically quiet, and C6: TGF-b dominant). We found that the expression level of PHF10 varied significantly among the five immune subtypes (p <0.01, **Figure 8A**). Next, we investigated the correlations between PHF10 expression and immune subtypes in human pan-cancer. Specifically, PHF10 expression was significantly correlated with immune subtypes in BLCA, STAD, LGG, BRCA, COAD, and KIRC (all p < 0.01, **Figures 8B–G**). The relationship between PHF10 and immune infiltration in PAAD and human pan-cancer was explored *via* the TIMER database. PHF10 expression was significantly associated with the content of immune cells in PAAD, including CD4+ T cells (R = 0.47), CD8+ T cells (R = 0.52), B cells (R = 0.35), neutrophils (R = 0.46), macrophages (R = 0.54), and dendritic cells (R = 0.48) (all p < 0.001, **Figures 9A–F**). Moreover, we further investigated the relationship between PHF10 and immune infiltration in human pan-cancer. The results showed that PHF10 mRNA level was associated with the immune infiltration in various types of cancer, including KICH, KIRC, SKCM, and THCA (all p < 0.05, R > 0.3, **Supplementary Figures 2A-L**). Overall, these findings strongly suggest that PHF10 plays an important role in tumor immunity.

## Discussion

Pancreatic cancer has a high morbidity and mortality across the world, and the treatment of PAAD still remains a challenge for human beings. Therefore, there is an urgent need to find new prognostic and therapeutic indicators to improve the survival of patients with PAAD. Recently, with the rapid development of bioinformatics, more and more novel biomarkers have been found, and eRNAs are one of them. eRNAs are a special subtype of non-coding RNAs, which are transcribed from the gene enhancer region and could regulate the expression of corresponding genes (6). Many studies demonstrated that the dysregulation of eRNA expression was associated with the occurrence of various human tumors (27, 28). Based on previous studies, we obtained a total of 1,559 eRNAs and its predicted targets. First, we identified a subset of eRNAs that significantly correlated with overall survival. Then, we further select the eRNAs that co-expressed with the target gene. The most survival-related eRNAs and its target genes were selected for further analysis. We found that LINC00242 was the most survival-related eRNAs and highly correlated with the predicted target PHF10.

In this study, we found that LINC00242 and its target gene PHF10 were significantly upregulated in PAAD tissues compared with that in normal tissues. Downregulation of LINC00242 showed a significant correlation with poor clinicopathological features, including higher histological grade, tumor recurrence, and advanced AJCC stage. Furthermore, we found that downregulated LINC00242 was significantly correlated with poor OS. All results indicated that LINC00242 had a tumor-suppressive effect in PAAD. Additionally, univariate and multivariate Cox analyses revealed that LINC00242 and PHF10 were associated with patients’ OS in PAAD, but only LINC00242 had an independent prognostic effect. Furthermore, we used external experiments to further study the regulatory relationship between LINC00242 and PHF10 in pancreatic cancer cell lines. LINC00242 was knocked down by transfection with siRNA. We found that PHF10 expression was significantly downregulated in the si-LINC00242 group compared with the si-control group. This suggests that PHF10 is a downstream target gene of LINC00242.

Pan-cancer analysis can find the similarity and difference of tumor, which is helpful in cancer prevention and treatment. Recently, many pan-cancer analyses demonstrated that gene mutation and RNA alterations were associated with the occurrence and development of cancer (29, 30). However, the function of LINC00242 and PHF10 in human pan-cancer is not clear. In the current study, pan-cancer analysis demonstrated that LINC00242 and PHF10 co-expressed in many types of cancer. This result suggested that there was a regulatory relationship between LINC00242 and PHF10. Interestingly, a high expression of LINC00242 was closely related to poor prognosis in KIRC and ACC, which was contrary to PAAD.

Since the function of LINC00242 is not yet clear, we tried to clarify its role by identifying its co-expressed genes. Then, a total of 1,640 genes were found to be significantly correlated with LINC00242. The enrichment analysis of GO and KEGG shows that LINC00242 was related to the immune-related processes. Based on these results, novel therapies that increase the expression of LINC00242 may help induce protective immunity to effectively treat PAAD.

The tumor microenvironment (TME) plays a key role in tumor immunotherapy and has attracted more and more attention from researchers in recent years. On the one hand, immune cells in the tumor microenvironment play an antitumor role by recognizing and killing cancer cells. On the other hand, tumor cells can avoid being killed by immune cells in a number of ways. CD4+ and CD8+T cells are an important part of the tumor microenvironment and kill tumor cells by exerting specific immune responses (31). M1 tumor-associated macrophages play an antitumor role, while M2 tumor-associated macrophages can promote tumor growth and metastasis (32). These results demonstrate that the immune microenvironment plays a critical role in tumor progression. Up to now, there are few studies on PHF10 and immune infiltration. We found that the mRNA level of PHF10 was significantly associated with the immune infiltration in various types of cancer. After further analysis by the CIBERSORT algorithm, we found that the levels of immune cell infiltration were significantly different between the PHF10-H and PHF10-L groups. These new findings have made substantial progress in determining the significant role of PHF10 in immune infiltration.

In this study, we use the unsupervised hierarchical clustering algorithm to divide the samples into three categories based on 29 immune cell types. Compared with the Immunity-M and Immunity-L groups, the expression of PHF10 was higher in the Immunity-H group, suggesting that elevated PHF10 level could recruit more immune cells. Additionally, PHF10 has a significant co-expression relationship with immune checkpoint genes CTLA-4 and PD-L1. It seems that high expression of PHF10 may be a candidate predictor of the efficacy of anti-PDL-1/CTLA4 therapy, and combination of PHF10 blockade and anti-PD-L1/CTLA4 mAb may be a potentially effective treatment for PAAD. As there are great differences in the sensitivity of different immune subtypes to immunotherapy, it is very important to correctly distinguish different immune subtypes for cancer immunotherapy. In this study, the expression of PHF10 varied significantly among the five immune subtypes in pancreatic cancer. The pan-cancer results further confirmed that there was a significant correlation between PHF10 and immunophenotype, indicating that PHF10 may have potential value in immunotherapy.

There are some limitations to this study. Firstly, further experiments are needed to verify the regulatory relationship between LINC00242 and its target genes PHF10. Second, the sample size and the clinical information were limited. Third, the prognostic efficacy of LINC00242 and PHF10 and their regulatory relationship should be verified in more pancreatic cancer datasets.

In conclusion, this study demonstrated that LINC00242 is a key survival-associated eRNA in PAAD. Downregulation of LINC00242 showed a significant correlation with poor clinicopathological features. Pan-cancer analysis further confirmed the prognostic value of LINC00242. PHF10 is a target gene of LINC00242 and has been shown to co-express with LINC00242 in a variety of cancers. External experiments further demonstrated that PHF10 is the downstream target gene of LINC00242. PHF10 expression was found to be significantly correlated with the immune cell infiltration and immune subtype across many cancer types. The results of this study provide a means for predicting the prognosis of patients with PAAD and a promising target for immunotherapy.

## Data Availability Statement

The original contributions presented in the study are included in the article/**Supplementary Material**. Further inquiries can be directed to the corresponding author.

## Author Contributions

WT conceived and designed the study. WT and LZ performed the experiments. WT and LZ wrote the manuscript. LZ, ZL, and LY revised the manuscript. YZ supervised the study. All authors contributed to the article and approved the submitted version.

## Funding

This work was supported by the Tianjin Natural Science Foundation (20JCYBJC01310), Tianjin Science and Technology Project (19ZXDBSY00010), Tianjin Health Science and Technology Project (ZC20218), and Tianjin Health Science and Technology Project (ZC20064).

## Conflict of Interest

The authors declare that the research was conducted in the absence of any commercial or financial relationships that could be construed as a potential conflict of interest.

## Publisher’s Note

All claims expressed in this article are solely those of the authors and do not necessarily represent those of their affiliated organizations, or those of the publisher, the editors and the reviewers. Any product that may be evaluated in this article, or claim that may be made by its manufacturer, is not guaranteed or endorsed by the publisher.

## Supplementary Material

Supplementary Figure 1 PHF10 mRNA expression overview in pan-cancer from TIMER database (https://cistrome.shinyapps.io/timer/).

Supplementary Figure 2 Correlation analysis between PHF10 expression and immune cell infiltration in pan-cancer. **(A-L)** The expression of PHF10 was positively correlated with the infiltration of immune cells in pan-cancer.

Supplementary Figure 3 Survival analysis of LINC00242 and its target gene PHF10 in ICGC-PAAD cohort. **(A, B)** Correlation analysis of LINC00242 expression **(A)** as well as PHF10 expression **(B)** and patient survival in PAAD. **(C, D)** Comparison of LINC00242 expression **(C)** as well as PHF10 expression **(D)** between pancreatic cancer tissues and normal tissues.

Supplementary Figure 4 The integrated analysis of PHF10 with immune infiltration in ICGC-PAAD cohort. **(A)** The enrichment levels of 29 immune-related gene sets were calculated *via* the ssGSEA method. **(B)** Analysis of the difference of immune cell abundance between the low and high PHF10 expression group. **(C)** The expression of PHF10 varied significantly among the three clusters.

## Abbreviations

PAAD, pancreatic adenocarcinoma; eRNA, enhancer RNA; OS, overall survival; TCGA, The Cancer Genome Atlas; PresSTIGE, Predicting Specific Tissue Interactions of Genes and Enhancers; GO, Gene Ontology; KEGG, Kyoto Encyclopedia of Genes and Genomes; AJCC, American Joint Committee on Cancer.
