# Supplementary figures and images for: Enhancer RNA LINC00242-Induced Expression of PHF10 Drives a Better Prognosis in Pancreatic Adenocarcinoma
Source: Front Oncol. 2022 Jan 20;11:795090. doi: 10.3389/fonc.2021.795090 (PMC8812487; doi:10.3389/fonc.2021.795090)

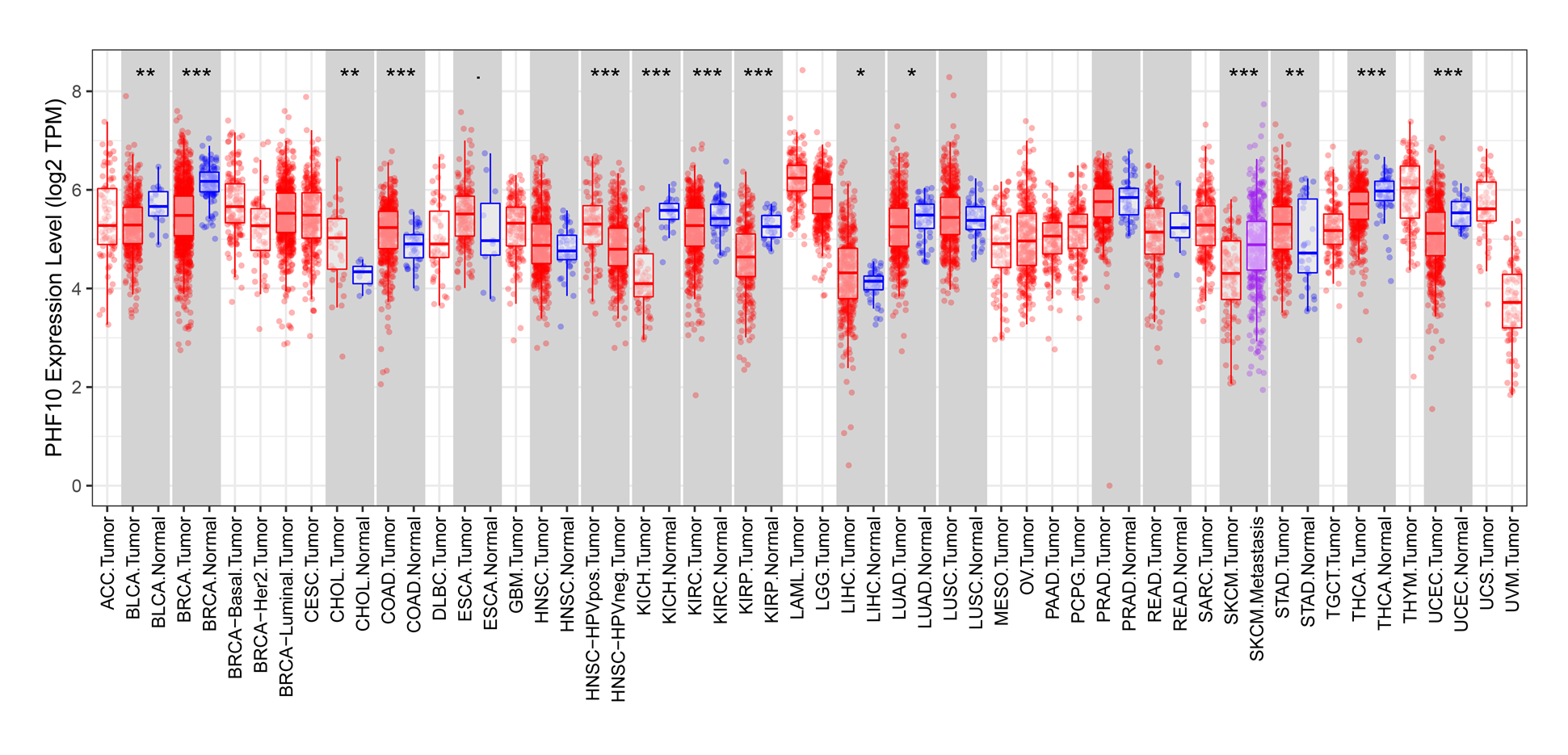

Supplement: Supplementary Figure 1 — PHF10 mRNA expression overview in pan-cancer from TIMER database (https://cistrome.shinyapps.io/timer/). [file Image_1.tif]

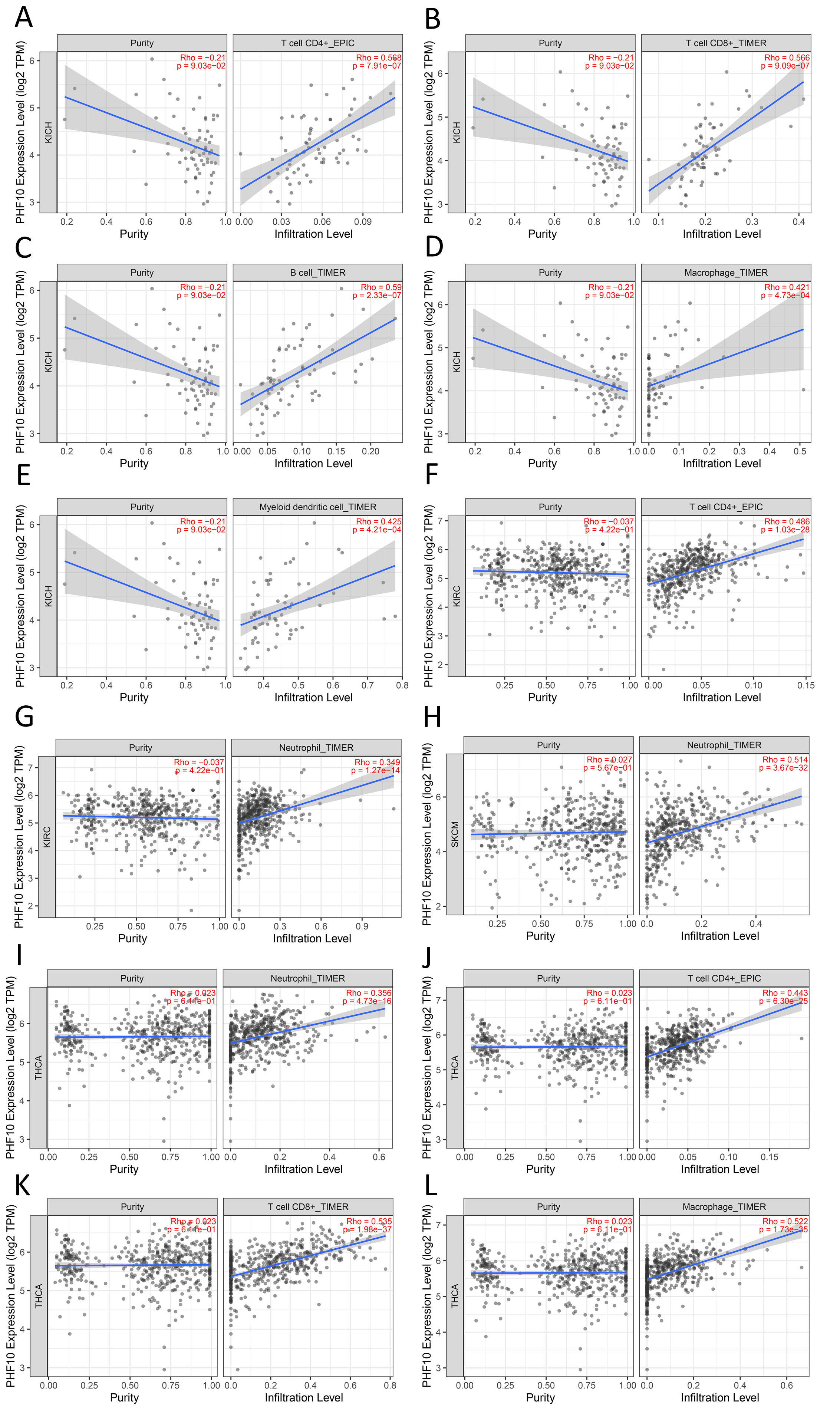

Supplement: Supplementary Figure 2 — Correlation analysis between PHF10 expression and immune cell infiltration in pan-cancer. (A-L) The expression of PHF10 was positively correlated with the infiltration of immune cells in pan-cancer. [file Image_2.tif]

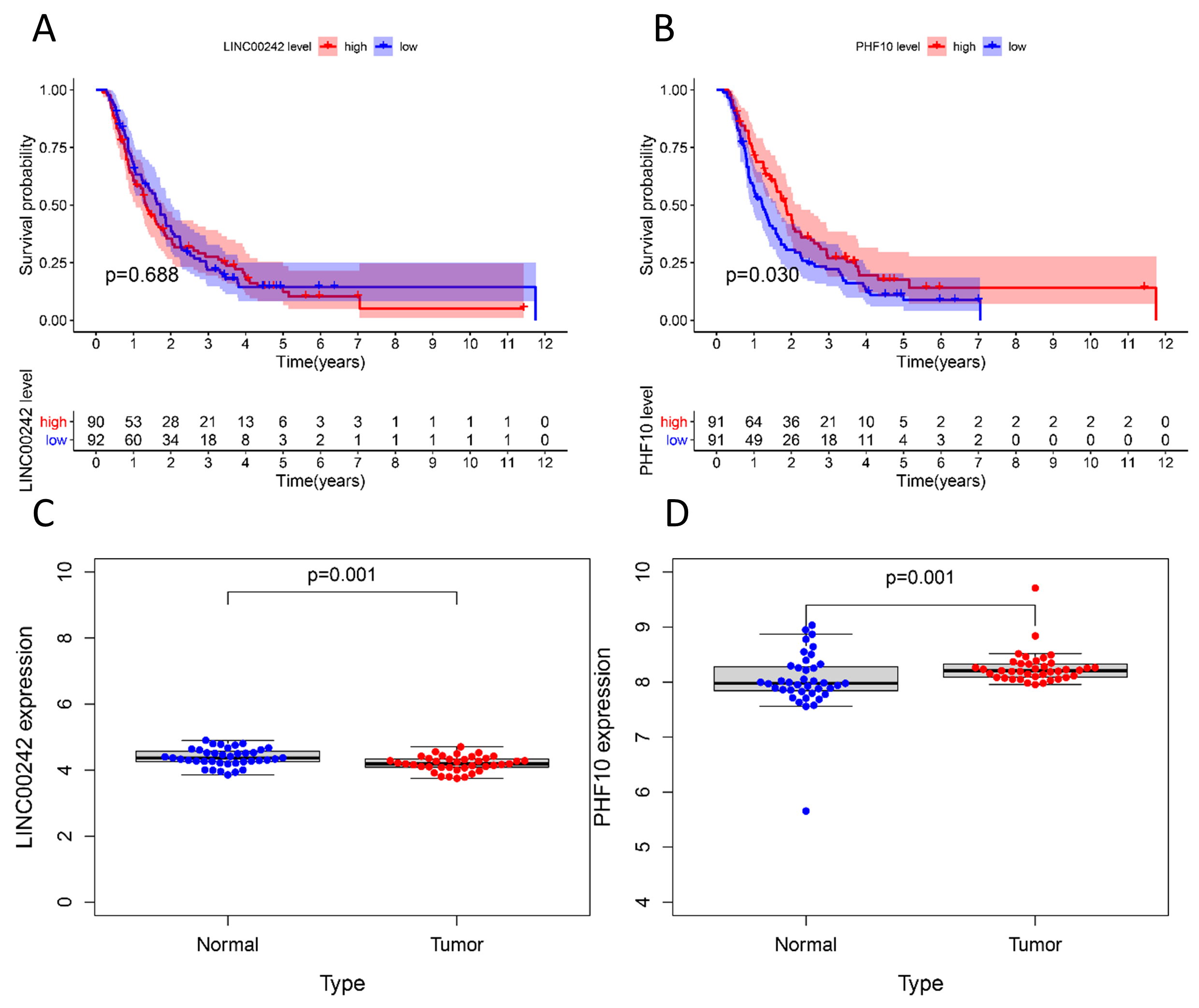

Supplement: Supplementary Figure 3 — Survival analysis of LINC00242 and its target gene PHF10 in ICGC-PAAD cohort. (A, B) Correlation analysis of LINC00242 expression (A) as well as PHF10 expression (B) and patient survival in PAAD. (C, D) Comparison of LINC00242 expression (C) as well as PHF10 expression (D) between pancreatic cancer tissues and normal tissues. [file Image_3.tif]

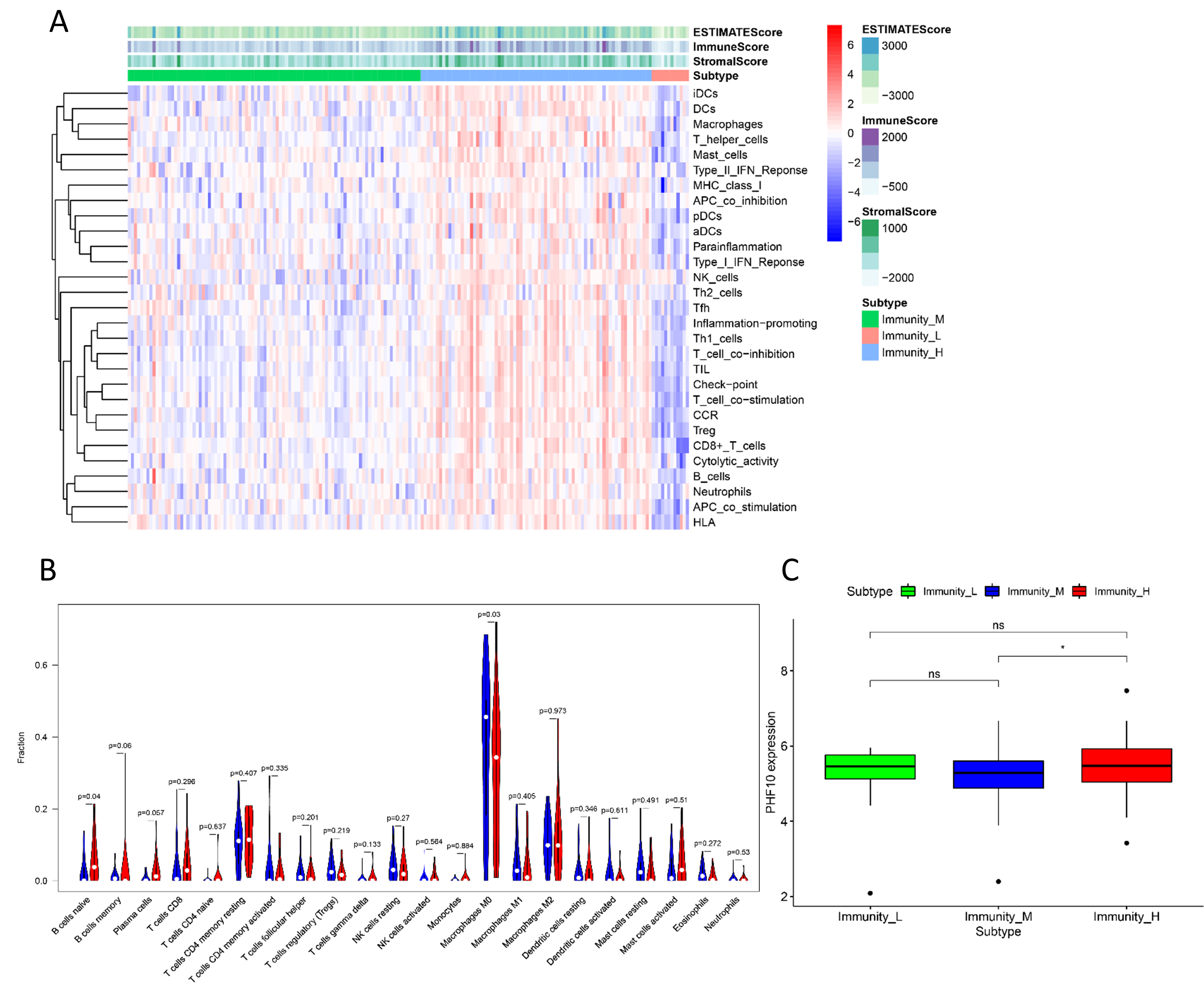

Supplement: Supplementary Figure 4 — The integrated analysis of PHF10 with immune infiltration in ICGC-PAAD cohort. (A) The enrichment levels of 29 immune-related gene sets were calculated via the ssGSEA method. (B) Analysis of the difference of immune cell abundance between the low and high PHF10 expression group. (C) The expression of PHF10 varied significantly among the three clusters. [file Image_4.tif]
