# Supplementary material for: Enhancer RNA LINC00242-Induced Expression of PHF10 Drives a Better Prognosis in Pancreatic Adenocarcinoma
Source: Front Oncol. 2022 Jan 20;11:795090. doi: 10.3389/fonc.2021.795090 (PMC8812487; doi:10.3389/fonc.2021.795090)
Supplement: Supplementary file 5 [file Table_1.docx]

|  | **Supplementary Table 1. Correlations between the expression of LINC00242 and clinicopathologic characteristics in PAAD.** | | | | | |
| --- | --- | --- | --- | --- | --- | --- |
| **Characteristic** | | **n (%)** | **LINC00242 Expression (%)** | | **χ^2^** | P |
|  |  |  | **High** | **Low** |  |  |
| Total | | 177(100) | 89 | 88 |  |  |
| Age | |  |  |  | 0.139 | 0.710 |
| ≤65 | | 93(52.5) | 48(53.9) | 45(51.1) |  |  |
| > 65 | | 84(47.6) | 41(46.1) | 43(48.9) |  |  |
| Gender | |  |  |  | 0.055 | 0.815 |
| Female | | 80(45.2) | 41(46.1) | 39(44.3) |  |  |
| Male | | 97(54.8) | 48(53.9) | 49(55.7) |  |  |
| Cancer status | |  |  |  | 10.540 | **0.005** |
| Tumor free | | 44(24.9) | 31(34.8) | 13(14.8) |  |  |
| With tumor | | 83(46.9) | 39(43.9) | 44(50) |  |  |
| Unknow | | 50(28.2) | 19(21.3) | 31(35.2) |  |  |
| Race | |  |  |  | 8.789 | **0.020** |
| Asian | | 11(6.2) | 1(1.1) | 10(11.3) |  |  |
| White | | 156(88.3) | 82(92.2) | 74(84.1) |  |  |
| Black | | 6(3.3) | 4(4.5) | 2(2.3) |  |  |
| Unknow | | 4(2.2) | 2(2.2) | 2(2.3) |  |  |
| AJCC stage | |  |  |  | 5.023 | 0.152 |
| Stage I | | 21(11.9) | 15(16.9) | 6(6.8) |  |  |
| Stage II | | 145(81.9) | 68(76.4) | 77(87.5) |  |  |
| Stage III | | 3(1.7) | 1(1.1) | 2(22.7) |  |  |
| Stage IV | | 5(2.8) | 2(2.2) | 3(3.4) |  |  |
| Unknown | | 3(1.7) | 3(3.3) | 0(0.0) |  |  |
| Grade | |  |  |  | 5.861 | 0.093 |
| G1 | | 30(19.9) | 20(22.5) | 10(11.4) |  |  |
| G2 | | 95(53.7) | 43(48.3) | 52(59.1) |  |  |
| G3 | | 48(27.1) | 23(25.8) | 25(28.4) |  |  |
| G4 | | 2(1.1) | 2(2.2) | 0(0.0) |  |  |
| Unknown | | 2(1.1) | 1(1.1) | 1(1.1) |  |  |
| history of chronic pancreatitis | |  |  |  | 8.210 | **0.004** |
| NO | | 127(71.8) | 64(71.9) | 63(71.6) |  |  |
| YES | | 16(9.0) | 2(2.3) | 14(15.9) |  |  |
| Unknown | | 34(19.2) | 23(25.8) | 11(12.5) |  |  |
| History of diabetes | |  |  |  | 0.013 | 0.910 |
| NO | | 107(60.5) | 49(55.1) | 58(65.9) |  |  |
| YES | | 38(21.5) | 17(19.1) | 21(23.9) |  |  |
| Unknown | | 32(18.1) | 23(25.8) | 9(10.2) |  |  |
