# Supplementary material for: Enhancer RNA LINC00242-Induced Expression of PHF10 Drives a Better Prognosis in Pancreatic Adenocarcinoma
Source: Front Oncol. 2022 Jan 20;11:795090. doi: 10.3389/fonc.2021.795090 (PMC8812487; doi:10.3389/fonc.2021.795090)
Supplement: Supplementary file 6 [file Table_2.docx]

| **Supplementary Table 2. Survival-associated eRNAs and their predicted target.** | | | | |
| --- | --- | --- | --- | --- |
| **eRNA** | **Log-rank test p-value** | **Target** | **Correlation coefficient r** | **Cor-Pvalue** |
| LINC00242 | <0.001 | PHF10 | 0.412 | <0.001 |
| AFG3L1P | <0.001 | GAS8 | 0.563 | <0.001 |
| AFG3L1P | <0.001 | MC1R | 0.734 | <0.001 |
| SSPOP | <0.001 | ATP6V0E2 | 0.510 | <0.001 |
| SSPOP | <0.001 | ZNF467 | 0.679 | <0.001 |
| SSPOP | <0.001 | ZNF862 | 0.648 | <0.001 |
| SSPOP | <0.001 | KRBA1 | 0.520 | <0.001 |
| LRRC8C-DT | 0.001 | LRRC8C | 0.606 | <0.001 |
| LINC02257 | 0.001 | DUSP10 | 0.408 | <0.001 |
| TAFA2 | 0.002 | USP15 | 0.404 | <0.001 |
| AC025539.1 | 0.002 | HS3ST1 | 0.533 | <0.001 |
| STK3 | 0.002 | NIPAL2 | 0.451 | <0.001 |
| TPT1-AS1 | 0.002 | SNORA31 | 0.553 | <0.001 |
| TMEM161B-AS1 | 0.004 | TMEM161B | 0.490 | <0.001 |
| ACYP2 | 0.004 | ACYP2 | 1.000 | <0.001 |
| B3GAT1-DT | 0.006 | B3GAT1 | 0.496 | <0.001 |
| WHAMMP2 | 0.006 | HERC2P9 | 0.535 | <0.001 |
| AC068580.2 | 0.008 | SYT8 | 0.459 | <0.001 |
| RASSF8-AS1 | 0.009 | RASSF8 | 0.511 | <0.001 |
