# Supplementary material for: Enhancer RNA LINC00242-Induced Expression of PHF10 Drives a Better Prognosis in Pancreatic Adenocarcinoma
Source: Front Oncol. 2022 Jan 20;11:795090. doi: 10.3389/fonc.2021.795090 (PMC8812487; doi:10.3389/fonc.2021.795090)
Supplement: Supplementary file 7 [file Table_3.docx]

| **Supplementary Table 3. Kaplan-Meier survival analysis and correlations analysis for LINC00242 and PHF10 in pan-cancer (33 types of cancer from TCGA).** | | | | |
| --- | --- | --- | --- | --- |
| **Abbreviation** | **Detail** | **Log-rank test p-value** | **Correlation coefficient r** | **Cor-Pvalue** |
| **ACC** | Adrenocortical carcinoma | 0.009 | 0.319 | <0.01 |
| BLCA | Bladder Urothelial Carcinoma | 0.378 | 0.122 | 0.013 |
| BRCA | Breast invasive carcinoma | 0.401 | 0.199 | <0.01 |
| CESC | Cervical squamous cell carcinoma and endocervical adenocarcinoma | 0.666 | 0.252 | <0.01 |
| CHOL | Cholangio carcinoma | 0.850 | 0.037 | 0.829 |
| COAD | Colon adenocarcinoma | 0.379 | 0.070 | 0.131 |
| DLBC | Lymphoid Neoplasm Diffuse Large B-cell Lymphoma | 0.183 | 0.359 | 0.013 |
| ESCA | Esophageal carcinoma | 0.267 | 0.310 | <0.01 |
| GBM | Glioblastoma multiforme | 0.482 | 0.281 | <0.01 |
| HNSC | Head and Neck squamous cell carcinoma | 0.840 | 0.156 | <0.01 |
| KICH | Kidney Chromophobe | 0.576 | 0.106 | 0.399 |
| **KIRC** | Kidney renal clear cell carcinoma | 0.009 | 0.032 | 0.462 |
| KIRP | Kidney renal papillary cell carcinoma | 0.102 | 0.098 | 0.097 |
| LAML | Acute Myeloid Leukemia | 0.929 | 0.177 | 0.029 |
| LGG | Brain Lower Grade Glioma | 0.733 | -0.039 | 0.375 |
| LIHC | Liver hepatocellular carcinoma | 0.713 | 0.446 | <0.01 |
| LUAD | Lung adenocarcinoma | 0.722 | 0.282 | <0.01 |
| LUSC | Lung squamous cell carcinoma | 0.756 | 0.234 | <0.01 |
| MESO | Mesothelioma | 0.367 | 0.199 | 0.066 |
| OV | Ovarian serous cystadenocarcinoma | 0.195 | 0.293 | <0.01 |
| **PAAD** | Pancreatic adenocarcinoma | 0.000 | 0.412 | <0.01 |
| PCPG | Pheochromocytoma and Paraganglioma | 0.776 | 0.226 | <0.01 |
| PRAD | Prostate adenocarcinoma | 0.854 | 0.014 | 0.755 |
| READ | Rectum adenocarcinoma | 0.891 | 0.125 | 0.108 |
| SARC | Sarcoma | 0.101 | 0.224 | <0.01 |
| SKCM | Skin Cutaneous Melanoma | 0.525 | 0.333 | <0.01 |
| STAD | Stomach adenocarcinoma | 0.633 | 0.300 | <0.01 |
| TGCT | Testicular Germ Cell Tumors | 0.079 | 0.661 | <0.01 |
| THCA | Thyroid carcinoma | 0.850 | 0.238 | <0.01 |
| THYM | Thymoma | 0.401 | 0.270 | <0.01 |
| UCEC | Uterine Corpus Endometrial Carcinoma | 0.434 | 0.090 | 0.034 |
| UCS | Uterine Carcinosarcoma | 0.050 | 0.565 | <0.01 |
| UVM | Uveal Melanoma | 0.105 | 0.443 | <0.01 |
