# Supplementary material for: Enhancer RNA LINC00242-Induced Expression of PHF10 Drives a Better Prognosis in Pancreatic Adenocarcinoma
Source: Front Oncol. 2022 Jan 20;11:795090. doi: 10.3389/fonc.2021.795090 (PMC8812487; doi:10.3389/fonc.2021.795090)
Supplement: Supplementary file 8 [file Table_4.docx]

| The summary of clinical characteristics for the two independent PAAD cohorts | | |
| --- | --- | --- |
| Factors | ICGC-PAAD | GSE15471 |
| Total | 182 | 36 |
| Age |  | - |
| ≤65 | 78 | - |
| > 65 | 86 | - |
| Unknown | 18 | - |
| Gender |  | - |
| Female | 83 | - |
| Male | 99 | - |
| AJCC stage |  | - |
| Stage I | 8 | - |
| Stage II | 114 | - |
| Stage III | 3 | - |
| Stage IV | 2 | - |
| Unknown | 55 | - |
| Grade |  | - |
| Moderately differentiated： | 57 | - |
| Well differentiated： | 20 | - |
| Poorly differentiated： | 41 | - |
| Unknown | 64 | - |
